# Conservative Treatment in Early Cervical Cancer

**Published:** 2013-09

**Authors:** Mojgan Karimi-Zarchi, Azamsadat Mousavi, Mitra Modares Gilani, Esmat Barooti, Ashrafosadat Miratashi-Yazdi, Atefe Dehghani

**Affiliations:** 1 Associate Prof, Gynecologist Oncologist, Shahid Sadoughi University of Medical Science, Yazd, Iran;; 2 Prof, Gynecologist Oncologist, Tehran University of Medical Science, Tehran, Iran;; 3 Associate Prof, Functional Gynecology, Taleghani Hospital, Velenjak Avenue, Tehran, Iran;; 4 Assistant Prof, Internal medicine, Yazd Branch, Islamic Azad University, Yazd , Iran;; 5 M.D, Shahid Sadoughi University of Medical Science, Yazd, Iran

**Keywords:** Cervical cancer, early stage, conservative, Quality of life, fertility preservation

## Abstract

Purpose of review: The aim of this study was to describe fertility preservation methods to improve quality of life of early stages of cervical cancer. Recent finding: Although definite treatment of early stages of cervical cancer including stages IA,IB1 and IIA non-bulky is radial hysterectomy, this method is used in perimenopousal period in which fertility preservation is not important. Whenever fertility preservation is so important, some methods like radical trachelectomy and laparoscopic lymphadenectomy are used to rule out lymphatic metastases. Summary: If any visible lesion on cervix is found, pelvic MRI is helpful and during operation, trachelectomy samples are sent for frozen section and margin study. Radical trachelectomy is done vaginal or abdominal. Overall relapse rate of cervical cancer in radical trachelectomy and radical hysterectomy is the same. Complications of radical trachelectomy include chronic vaginal discharge, abnormal uterine bleeding, dysmenorrhea, inflammation and ulcer due to cercelage, amenorrhea, cervical stenosis and pregnancy complications following trachelectomy including 2^nd^ trimester abortion and premature labor following cervical prematurity.The best and preferred method of labor is cesarean section. Neoadjuant chemotherapy followed by radical trachelectomy in large cervical lesions is a suitable treatment. Ultraconservative operations like large cold knife conization, simple trachelectomy with laparoscopic lymphadenectomy and sentinel lymph node mapping are suitable for very small lesions.

## INTRODUCTION

Cervical cancer is the 3^rd^ common cancer of genitalia system in women following endometrial and ovarian cancers in USA (1) and 2^nd^ common gynecological cancer following endometrial or ovarian cancer in some parts of Iran (2).

It should be noted that in some developing countries, cancer of cervix is the most common gynecological cancer due to lack of screening programs (1, 2).

42% of cervical cancer is diagnosed in women younger than 45. Regarding fertility preservation, conservative management in early stages of cervical cancer especially in cases with survival rate higher than 90%, is very important (3, 4).

Conservative management has done during previous decades have had good outcomes. Early stages of cervical cancer include IB1, IA1 and squamous cell type of non-bulky stage IIA1 (5).

Multiple options of fertility preservation in early stages of cervical cancer, outcomes of pregnancy and complications of treatment are discussed in our study.

### Operation

Multiple surgical operations to treat cervical cancer are recommended. In some special conditions, fertility preserving methods can be used.

**Abdominal Radical Hysterectomy (ARH).** Radical Hysterectomy with pelvic and Para aortic lymphadenectomy has similar efficacy in comparison with chemo radiation, but morbidity and complications of these methods in managing early stages of cervical cancer are different.

Ovarian function preservation in young women is possible with radical hysterectomy in spite of chemoradiation. Shortening of vagina following chemoradiation and dyspareunia lead to worsening of quality of life too (6).

**Laparoscopic surgery.** Minimally invasive radical hysterectomy and/or laparoscopically assisted radical vaginal hysterectomy lead to less surgical complications and better quality of life during last decades.

Rate of complications of radical vaginal hysterectomy and laparoscopic lymphadenectomy was similar to abdominal hysterectomy in a study done on 200 cases of stage IA1 with lymphatic or lymphovascular invasion to stage IIB (7).

### Robotic-assisted Laparoscopic Radical Hysterectomy.

**Conservative surgery.** In women who preferred fertility preservation and tumoral factors inhibit non radical surgery, in stage IA1 without lymphovascular involvement (LVSI), conization is possible. Studies done on 149 stage IA1 cases younger than 40, showed that survival rate of conization is similar to hysterectomy (8).

In stage IA2 tumors with invasion less than 5 mm and horizontal spread less than 7 mm, conization with pelvic lymph node dissection is indicated because lymph node metastases is about 5-8 % in these cases (9).

**Pregnancy following conization.** If section and length of cone is less than 18 mm and 15 mm respectively, conization has no side effect on pregnancy, but if section and length of cone is over 18 mm and 15 mm respectively, rate of premature labor and Premature Rupture of Membrane (PROM) are up to 25% and 15% respectively (10).

### Vaginal Radical Trachelectomy(VRT) and its indications

Since 1980, vaginal radical trachelectomy and laparoscopic lymphadenectomy has been introduced by Daniel Dorgent. Inclusion criteria of this management are as below:

1. Desire to preserve fertility;
2. Age younger than 40 or 45;
3. Stage IA1 with horizontal involvement;
4. Stage IB1 with diameter less than 2-2.5 cm and intact cervical nodes;
5. Squamous or adenocarcinoma types;
6. No wide lymphovascular involvement (LVSI);
7. Intact lymph nodes;
8. No high risk pathology of cervical cancer (11).

To achieve this goal, MRI and careful studies are helpful in correct staging.

About 40% of cases candidate for radical hysterectomy can undergo VRT. It should be noted that about 12% these cases may need adjuvant radiotherapy and radical hysterectomy during VRT due to positive frozen section or involved endocervical margins (12).

Patients Selection: Tumor size, site, severity of canal of cervix involvement, length of cervical canal and distance between upper margin of lesion and uterine isthmus. Also pelvic MRI is helpful in patients’ selection.

If distance between upper margin of the lesion and isthmus is less than 1 cm, neoadjuvant chemotherapy followed by VRT is preferred (13).

The intent of the radical abdominal trachelectomy is to resect the cervix, upper 1-2 cm of the vagina, parametrium, and paracolpos in a similar manner to a type III radical abdominal hysterectomy but sparing the uterine fundus or corpus. The procedure is begun by developing the paravesical and Para rectal spaces and dissecting the bladder caudal to the mid vagina. The round ligaments are divided and large Kelly clamps are placed on the medial round ligaments to manipulate the uterus. Care is taken not to destroy the cornea or the utero-ovarian pedicles. The infundibulopelvic ligaments with ovarian blood supply are kept intact. Care is also taken not to injure the fallopian tubes or disrupt the uteroovarian ligament. The lower uterine segment is then estimated, and clamps are placed at the level of the internal os. Using a knife, the radical trachelectomy is completed by separating the fundus from the isthmus or upper endocervix at approximately 5 mm below the level of the internal os, if possible. It’s not definite which sutures are suitable for cerclage, but evidences show that monophylamen polypropylene #0 suture can be a good choice due to good elasticity and lower risk of infection. The elasticity of sutures should not be so much not to damage stroma of the cervix (14, 15).

Anyway, traditional cerclage is preferred and alternative methods should be considered for unsuccessful operations (16). A study done on 140 candidates of VRT showed that 10% of operations were canceled due to lymphatic involvement.

### Importance of frozen section during operation

If a visible lesion is present on clinical evaluation, we send the specimen for frozen section. The pathologist is asked to perform the frozen section longitudinally (from the exocervix to the endocervix) at the level of the lesion in order to determine the distance between the upper endocervical tumor margin and the specimen excision margin. Ideally, 8 to 10 mm of normal tissue should be present; otherwise an additional section of the cervix may need to be removed. If the tumor extends higher into the endocervical canal and the margins remain positive or too close (<5 mm), it may be necessary to perform a complete radical hysterectomy (17).

If no visible lesion is present on clinical evaluation, (e.g., after a diagnostic cone), we prefer to keep the trachelectomy specimen intact for final pathological analysis.

### VRT complications vs. hysterectomy

Morbidity and complications of VRT is equal or less than radical hysterectomy. In a largest survey showed that morbidity during laparoscopic-assisted vaginal radical trachelectomy was comparable to laparoscopic-assisted vaginal radical hysterectomy in 118 cases (18).

Rate of complication during operation is the same in both groups. (2.5% vs. 1.8% respectively) Post-operative complications were 21.2% and 19.4% respectively. The findings were the same in other studies too (19-21).

In another study, morbidity rate during VRT was less than ARH, but long term post-operative complications were higher in VRT due to cerclage sutures and uterine preservation.Typical complications reported after radical trachelectomy include dysmenorrhea (24%), dysplastic Pap smears (24%), metrohrragia (17%), problems with cerclage sutures (14%), excessive vaginal discharge (14%), isthmic stenosis (10%), amenorrhea (7%), and occasional reports of deep dyspareunia. (12)

This method can make problems in fertilization *in vitro.* (22)

### Post-operative complications

Relapse and mortality rates are less than 5% and 2-3% respectively (23) which is equal to vaginal hysterectomy. 25% of relapses occur in pelvic-Para aortic and supraclavicular lymph nodes (24). 40% of relapse rate occurred in parametrium or pelvic walls that probably might be due to incorrect incision of parametrium or microscopic LVSI involvement. (25)

A model using tumor size and serum SCC Ag level is highly predictive of parametrial involvement in patients with stage IB1 cervical cancer and may identify candidates for less radical parametrial resection (26).

Risk factors of relapse are tumor size >2 cm (27), lymphovascular involvement (28, 29), non-squamous pathology (15, 18, 23) and massive invasion of tumor (29).

### Adjuvant therapy indications

Ten percent of VRT candidates need neoadjuvant therapy due to involved lymph nodes, positive margin and parametrium involvement in final pathology (23).

Although findings about relapse rate in involved lymph nodes or margin less than 5 mm are different and many of the cases who rejected to receive neoadjuvant radiotherapy didn’t have relapse and stayed alive (18, 30).

Post VRT follow up by cervical cytology and colposcopy should be done every 3-4 months in first 3 years, every 6 months in next 2 years and then annually (31, 32).

In abnormal Pap smear following VRT, well colposcopy is needed, but regarding abnormalPap smear in many relapses, each abnormal Papsmear should be monitored well. Sometimes, HPV typing following trachelectomy can be helpful. In high risk HPV, prevalence of local relapse in higher and vaccination is recommended.

### Recommended time of hysterectomy

After completing child bearing, hysterectomy can be recommended. Complications of not doing hysterectomy have not been studied yet, so it’s not so routine to do it (19) and even C/S hysterectomy should be done in indicated conditions.

### Pregnancy following VRT

It should be discussed with candidate patients during preoperative consultation (29, 33, 34). Prevalence of infertility following VRT is 14-41 % (35).

Patients who have undergone radical trachelectomy may face problems conceiving naturally and may request assisted conception. In a cohort study, 12 to 15 women with gynecological problems became pregnant following VRT. 40-70% of problems is due to cervical stenosis and the rest is due to conditions unrelated to VRT (29, 34, 36).

### Pregnancy outcome

The majority (66%) of 200 women who underwent VRT had pregnancy. Of these, 12.5% ended prematurely at <32 weeks gestation, 25% delivered between 32 and 36 weeks and 42% delivered at term (>37 weeks) (16, 29, 37).

Preservation of childbearing function is a great advantage for patients with early-stage cervical cancer. Many patients do not seek parenthood immediately. First trimester miscarriage rate following VRT is about 16-20% (38). Cerclage often doesn’t inhibit abortion. In missed abortions, misoprostol can induce uterine contractions, but medical therapy is not so effective and D/C is a good alternative.We see no impairment of fertility and have solid data on pregnancy outcome. Premature labor is the main problem in pregnancy after RVT (39).

Second trimester miscarriage rate following VRT is higher than general population (9.5% vs. 4%) (16, 39). PROM due to chorioamnionitis can lead to premature labor (39).

To prevent premature labor due to shortening of cervix and premature function of it, it’s better to leave at least 1 cm of endocervix during trachelectomy which doesn’t increase rate of local relapse (39).

### Best Time of Pregnancy following VRT

It’s recommended to postpone pregnancy to at least 6 and preferably 12 months following VRT (16, 39). Perinatal care, screening and following up of genital tract infections, prophylactic antibiotics, bed rest and decreasing physical activity and routine prescription of steroids until lung maturity to decrease complications of premature labor are highly recommended (34).

However; in some studies physical activity restriction is just recommended in women with shortening of cervix, so serial measuring of length of canal of cervix can be helpful (34).

As prophylactic antibiotics couldn’t prevent premature labor in other conditions, there is not general agreement about prophylactic antibiotic in women with intact amniotic membrane.

If rupture of membrane occurred, termination of pregnancy is recommended in chorioamnionitis or pregnancy with at least 32-34 gestational ages, If not cerclage and prophylactic antibiotics continue (16, 34).

C/S is preferred following VRT because due to shortening of cervix, probability of lateral rupture of cervix is higher in vaginal labor, so C/S is recommended in 37-38 gestational age of pregnancy. Type of C/S incision is low transverse, but sometimes low vertical incision is used to decrease probability of lateral uterine damage and uterine artery laceration (19).

### Abdominal Radical Trachelectomy (ART)

This technique was described by Smith in 1997 for the first time (4). Radical trachelectomy as a new technique to preserve fertility in early stages of cervical cancer seems to be less effective in adenocarcinoma than squamous cell carcinoma due to a worse prognosis.

RVT looks as if it is a valid uterus-conserving surgery for women of reproductive age who have early-stage cervical carcinoma. However, in order to reach a final conclusion about the oncological and obstetrical results, further studies are needed with larger sample sizes and longer follow-up periods.

Abdominal radical trachelectomy is feasible and can be performed safely in a developing country in well-selected patients with early cervical cancer who wish to preserve their fertility. An alternative to the laparoscopic or vaginal approach is abdominal radical trachelectomy. In this approach the retroperitoneum is entered after the round ligaments have been divided, and a complete lymphadenectomy is performed. The uterine vessels are ligated at their origins and the uterine corpus is transected at the level of the internal os. The cervix is removed together with the parametria and the upper third of the vagina. After the placement of a cerclage, the proximal vaginal margins are sutured to the margins of the retained uterine body (15).

### Neoadjuvant Chemotherapy

For lesion larger than 2 cm,neoadjuvant chemotherapy with Taxol, ifosfamide, cis platin (ITP) is indicated. For decreasing of complication of chemotherapy on ovarian function (43-45). GnRH analogs can be helpful. Cryopreservation of oocyte or ovarian tissue in particular condition is useful (46).

### Ultraconservative procedure for fertility preservation

Large cold knife conization, simple trachelectomy with laparoscopic lymphadenectomy and sentinel lymph node mapping are suitable for very small lesions (47).

## CONCLUSION

Although radical hysterectomy with pelvic and para-aortic lymphadenectomy is the best treatment of early cervical cancer, but for some particular conditions we can do conservative management. They are including radical trachelectomy for stage IA1 and nun-bulky IB1, cold knife conization and simple trachelectomy with laparascopic lymphadenectomy for very small lesions. Selection of suitable patients for conservative treatment is the best important point for getting a good result without any recurrence or complication.
